# Selective Inhibitory Control in Middle Childhood

**DOI:** 10.3390/ijerph18126300

**Published:** 2021-06-10

**Authors:** Irene Rincón-Pérez, Alberto J. Sánchez-Carmona, Susana Arroyo-Lozano, Carlos García-Rubio, José Antonio Hinojosa, Alberto Fernández-Jaén, Sara López-Martín, Jacobo Albert

**Affiliations:** 1 Facultad de Psicología, Universidad Autónoma de Madrid, 28049 Madrid, Spain; irene.rincon@uam.es (I.R.-P.); garciarubio.carlos@gmail.com (C.G.-R.); sara.lopez@neuromottiva.com (S.L.-M.); 2 Unidad de Cartografía Cerebral, Instituto Pluridisciplinar, Universidad Complutense de Madrid, 28040 Madrid, Spain; asanchezcarmona@neuromottiva.com (A.J.S.-C.); hinojosa@pluri.ucm.es (J.A.H.); 3 Centro Neuromottiva, 28016 Madrid, Spain; susana.arroyo@neuromottiva.com; 4 Facultad de Psicología, Universidad Complutense de Madrid, 28223 Madrid, Spain; 5 Centro de Ciencia Cognitiva—C3, Universidad Antonio de Nebrija, 28015 Madrid, Spain; 6 Department of Pediatric Neurology, Hospital Universitario QuirónSalud, 28233 Madrid, Spain; aferjaen@telefonica.net; 7 School of Medicine, Universidad Europea de Madrid, 28670 Madrid, Spain

**Keywords:** executive control, selective stopping, strategies, SSRT, middle childhood

## Abstract

The main aim of this study was to investigate the development of selective inhibitory control in middle childhood, a critical period for the maturation of inhibition-related processes. To this end, 64 children aged 6–7 and 56 children aged 10–11 performed a stimulus-selective stop-signal task, which allowed us to estimate not only the efficiency of response inhibition (the stop-signal reaction time or SSRT), but also the strategy adopted by participants to achieve task demands. We found that the adoption of a non-selective (global) strategy characterized by stopping indiscriminately to all stimuli decreased in older children, so that most of them were able to interrupt their ongoing responses selectively at the end of middle childhood. Moreover, compared to younger children, older children were more efficient in their ability to cancel an initiated response (indexed by a shorter SSRT), regardless of which strategy they used. Additionally, we found improvements in other forms of impulsivity, such as the control of premature responding (waiting impulsivity), and attentional-related processes, such as intra-individual variability and distractibility. The present results suggest that middle childhood represents a milestone in the development of crucial aspects of inhibitory control, including selective stopping.

## 1. Introduction

Middle childhood is a critical phase of human development, spanning a period of time from roughly 6 to 11 years of age. It is characterized by marked changes in attention and executive functions, with widespread implications for later development [1,2,3]. In general, the development of these abilities is accompanied by pronounced brain maturation [4,5], such that by middle childhood, the frontoparietal and cingulo-opercular networks activated in executive function tasks are similar to those in adults, even if there are further quantitative changes in activity [6]. These cognitive processes are essential for achieving set goals and adapting to an environment in constant change, and previous research shows that they develop during middle childhood [7,8,9,10,11,12].

Inhibitory control refers to the suppression of goal-irrelevant stimuli, thoughts and behaviours. Thus, it is one of the basic executive functions needed to achieve set goals, and includes several aspects ranging from attentional inhibition to the ability to stop motor responses that are planned or already initiated [13,14,15]. This response inhibition may be conceptualized as global or selective depending on its complexity and the number of stimuli and responses involved. Global inhibition involves the suppression of all motor output, usually measured as the inhibition of a single motor response in reaction to a given stimulus. In contrast, selective inhibition is a more complex form of inhibitory control that is required in real world situations where either certain responses must be stopped but not others (response-selective stopping) or motor responses must be inhibited to certain stimuli but not others (stimulus-selective stopping) [16,17]. Selective inhibitory control requires the activation of some of the same brain areas needed for global stopping such as the inferior frontal gyrus and the pre-supplementary motor area [14,17], but also needs further activation of other regions including the dorsolateral prefrontal cortex and striatum [17,18,19]. This is possibly because of its greater complexity and the greater involvement of processes outside pure inhibition such as perceptive and attentional discrimination or working memory.

Inhibitory control can also be conceptualized as reactive or proactive [17,20]. Proactive inhibition refers to how knowing that there may be an upcoming response that should be cancelled affects performance in the task. Thus, it is goal-directed and denotes the inhibitory effort exerted in anticipation of stopping (before stop signals actually appear). By contrast, reactive inhibition is stimulus-driven, triggered by the presentation of the stop signal. Remarkably, although they are often presented as different dimensions of inhibitory control, proactive and selective inhibition are thought to be closely related. When faced with situations requiring selective stopping, individuals have goals regarding which response tendencies to control. This necessarily involves preparation before the stopping itself takes place [21]. Indeed, it seems that a more pronounced proactive control enhances selectivity in the execution and inhibition of motor responses [22,23].

Selective inhibitory control can be assessed using a stimulus-selective stopping task. In this kind of task, participants are instructed to respond as fast as possible to the go stimulus, and to stop their responses whenever a stop stimulus is presented shortly after the go stimulus in some trials (after what is called the stop signal delay or SSD). Selectivity is introduced into this task through another stimulus (the ignore or continue stimulus). This ignore stimulus is also presented just after the go stimulus in some trials, and indicates that the participant should finish the response that has likely begun as in a regular go trial. Theoretically, in this paradigm, participants are first required to discriminate between stop and ignore trials, and then cancel their responses selectively only after stop stimuli. However, recent behavioural and neural evidence in adults indicates that not all participants solve this task equally [16,18,24]: some complete the stopping process after discriminating the stop and ignore stimuli (Independent Discriminate then Stop-iDtS-and Dependent Discriminate then Stop-dDtS-strategies), but others stop indiscriminately to all stimuli and then resume their response when needed, if the stimulus was an ignore one (Stop then Discriminate-StD-strategy).

The stimulus-selective stopping task also allows for the calculation of the stop-signal reaction time or Stop-Signal Reaction Time (SSRT) as a measure of the latency of response inhibition both for global and selective implementations of inhibitory control (under the same conditions, a shorter SSRT suggests a better ability to inhibit). Unlike in the selective strategies, under the StD strategy, the stopping is global (not selective), and thus, it does not include the time needed to discriminate between stop and ignore signals. The SSRT is calculated based on the theoretical postulates of the horse race model [25], which assumes stochastic independence between the go and stop process [26]. In this framework, it is worth noting that the SSRT can be reliably estimated in participants using the iDtS and StD strategies [16], while SSRT estimation in the dDtS is subject to particular assumptions because the requirement to discriminate between stop and ignore signals generates a dependency between processes. Therefore, in this strategy, the independence assumption of the model is violated [16,27].

In addition to the SSRT and the strategies, the stimulus-selective stopping task offers the possibility to calculate other measures related to inhibitory control such as response adjustments after stop- and ignore-signal presentation and premature or anticipatory responses (responses emitted before the onset of the go stimulus). Although there are different interpretations about why individuals slow down after they commit an error and detect conflict [28,29,30], there is some consensus that this phenomenon involves cognitive control processes [28]. Within this framework, post-stop error and post-ignore slowing occurring within the stimulus-selective stopping task could be placed somewhere along the proactive–reactive control continuum. Although they are triggered by an external signal (stop or ignore), they both involve the activation of proactive control processes aimed towards preventing further errors and/or resolving conflict. On the other hand, premature responses denote waiting impulsivity or the inability to wait to emit responses until the stimulus appears, as opposed to stopping impulsivity or the inability to stop a response once it is required [31,32,33]. These two aspects of impulsivity are distinct but related, and are thought to be mediated by partially distinct neural networks [34].

While it is clear that inhibition in a broader sense develops during middle childhood [7,12,35], selective inhibition has been much less investigated. Some studies provide tangential evidence about the possible development of selective inhibitory control: there is some evidence of longer SSRTs for younger children at this stage (6–8 years) using tasks that involve a selective cancellation of the response [12,36,37]. However, these studies did not take into consideration the effect of the existence of different behavioural strategies on SSRT estimation, nor did they explore possible changes in the use of these strategies during middle childhood. Furthermore, attentional processes are also needed to successfully perform a stimulus-selective stop-signal task, because participants need to engage attentional processes in order to sustain performance throughout the task. Weaker attentional-related abilities result in distractibility and attentional lapses, which can be captured through the ex-Gaussian analysis of reaction times distributions and through the number of times the participant misses a response that should have been emitted [38,39,40]. Therefore, we also aimed to examine the development of attention in middle childhood, since it has been shown that this period is also critical for the improvement of attention-related functions [8,9,11].

The main aim of this study was to investigate the development of selective inhibitory control in middle childhood using a stimulus-selective stop-signal task, by comparing a group of children at the beginning of middle childhood (aged 6–7) to a group of children at the end of this developmental period (aged 10–11). Given that selective inhibition is more complex and requires the participation of more brain areas than non-selective or global inhibition [16,17,18,19,41], we hypothesized that selective stopping strategies (iDtS and dDtS) would be chosen by older children to a greater extent than by younger children. Moreover, we expected to find a greater efficiency of the stopping process (indicated by shorter SSRTs) in older children compared to younger children, even after taking into account the different strategies that participants adopt to solve the task. We also hypothesized reductions in premature responding, whereas hypotheses regarding post-signals adjustments were unclear due to the mixed results in previous literature at this developmental stage [42,43,44,45]. Finally, regarding attentional processes, we expected to find a reduction in intra-individual variability of reaction times [46,47] and in omission errors in older children.

## 2. Materials and Methods

### 2.1. Participants

One hundred and forty-three typically developing children with no history of psychiatric, neurological or sensory impairment were recruited from two local regular schools. Twenty-three participants were excluded due to one or more of the following reasons: the strategy used to complete the stimulus-selective stop-signal task could not be estimated (*n* = 21), the probability of responding on the stop trials (respond|signal) was higher than 0.75 (*n* = 5) [48] and/or there was a high probability of omissions (>30%; *n* = 2) [48]. Therefore, the final sample consisted of 120 participants: 64 younger children (age range 6–7 years, mean age = 6.47 ± 0.5) and 56 older children (age range 10–11 years; mean age = 10.21 ± 0.42). None of the participants had repeated a grade, nor did they need any curriculum adaptations. Gender distribution did not differ between groups (chi-squared test; χ^2^ = 0.15, df = 1, *p* = 0.7).

### 2.2. Stimulus-Selective Stopping Task

**Figure 1:**
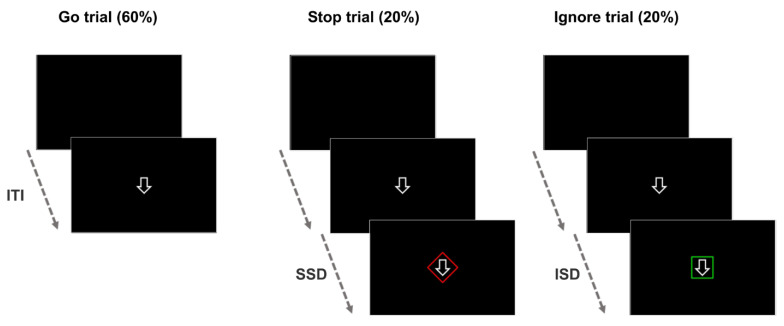
Schematic representation of the stimulus-selective stopping task. ms = milliseconds; ITI = inter-trial interval; SSD = stop-signal delay; ISD = ignore signal delay.

Participants completed a stimulus-selective stop-signal task adapted for children [18,19,24], where three different stimuli were presented (Figure 1). The go stimulus was a white down-pointing arrow on a black background, with a duration of 1500 ms. This arrow appeared on every trial and children were instructed to press the spacebar on a keyboard with the index finger of their dominant hand as soon as they saw it. Trials where only this go stimulus appeared (without being followed by any other stimuli) are called go trials and comprise 60% of the total trials. On another 20% of the trials, the go stimulus was followed by the appearance of the stop signal (a red diamond appearing around the go stimulus after the SSD). These trials are called the stop trials and children were told to stop their response when the red diamond appeared. Lastly, in the remaining 20% of the trials, the go stimulus was followed by the ignore stimulus (a green square that likewise appeared around the go stimulus after a short delay). In these trials, called the ignore trials, children were told to press the spacebar even if they saw the green square and finish their response as if nothing had happened and it was a regular go trial. Bright colours were used because salient stop signals such as these reduce the influence of perceptual discrimination processes on the SSRT, and therefore, the probability that group differences may be attributed to them is also reduced [19,41,48]. Furthermore, salient signals promote the use of a selective stopping strategy as opposed to a global non-selective strategy [41]. The order of trials was fully randomized.

These instructions were given to children before the task began, both on the computer screen and also orally. We emphasized the need to respond as fast and accurately as possible on go and ignore trials, and be as accurate as possible on stop trials. Children were also reminded that they should not wait for the square or diamond to appear, emitting their responses as soon as possible [48].

Each trial began with a black screen (the inter-trial interval or ITI) presented for either 500 or 1000 ms (with equal probability), and then, the go stimulus was always presented (1500 ms). Taking the ITI into account, each trial lasted either 2000 or 2500 ms in total. After the go stimulus, in stop trials, the stop signal was presented after a variable delay (SSD) that initially lasted for 250 ms, and was adjusted dynamically with a staircase tracking procedure from one stop trial to the next based on each child’s performance in order to achieve 0.5 probability of responding to a stop signal. After a successful inhibition, the SSD was increased (+50 ms), which decreased the probability of interrupting the response in the next stop trial. If inhibition was not successful and a response was emitted in the last stop trial, the next SSD decreased (−50 ms) so that the probability of stopping in the next stop trial increased. In ignore trials, the ignore stimulus was presented after the ignore signal delay (ISD), which was initially fixed to 250 ms but was always equated in subsequent trials to the most recent SSD with no adaptive adjustment. Overall, the task consisted of 230 trials (138 go trials, 46 stop trials and 46 ignore trials) all in one block that lasted 8.6 min.

Participants also carried out a short practice block of 30 trials (with the same characteristics described above) to ensure that they understood task instructions properly. The task was designed and implemented in MATLAB using Psychtoolbox Version 3 (www.psychtoolbox.org, accessed on 29 April 2021).

### 2.3. Task-Related Measures

#### 2.3.1. Inhibitory Control-Related Measures

Type of inhibitory control strategy (global vs. selective). The strategy used by each participant to solve the stimulus-selective stopping task was identified through comparison (independent t tests) of their mean go RT with ignore RT and failed stop RT, according to the method proposed by Bissett and Logan [16]. As mentioned previously, this results in three different possible strategies. The iDtS is characterized by a failed stop RT faster than go RT, and an ignore RT not different from go RT. The dDtS strategy features a failed stop RT not different from go RT, and an ignore RT slower than go RT. Lastly, the StD strategy is identified by a failed stop RT faster than go RT, and an ignore RT slower than go RT. Subsequently, each t value was transformed into a Bayes factor (BF) to compare the evidence for and against the null hypothesis without bias [49]. A BF represents a ratio between the likelihood of data given the null hypothesis and the likelihood of data given the alternative hypothesis. We used Rouder’s Bayes factor calculator on the Perception and Cognition Lab website (http://pcl.missouri.edu/bf-two-sample, accessed on 29 April 2021) to convert t values and sample sizes into BFs. The Jeffrey–Zellner–Siow prior test was used due to the lack of any a priori assumptions [49]. A BF of 1 meant that there was no difference between RTs, and a BF ≠ 1 indicated that there was a difference.

SSRT. The SSRT represents the amount of time taken to cancel the already initiated response once the stop signal has been presented. We computed SSRTs using the integration method, given that this approach is less susceptible to distortion from skew in the go RT distribution and strategic slowing than the traditional mean method [50]. As mentioned in the introduction, the different strategies call for slightly different calculations of the SSRT. The stopping and the responding processes are supposed to be independent according to the horse race model [25]. Taking into account this framework, in the StD and the iDtS strategies, where processes remain independent and the assumptions of the horse race model are not violated, we use the integration method to calculate the SSRT based on the underlying go RT distribution. However, in the dDtS, there is a dependence between going and discriminating stop and ignore signals, and thus, the SSRT cannot be estimated using the go RT distribution, as Bisset and Logan [16] noted. These authors further proposed that if go RT was equally slow on both stop and ignore trials, the SSRT could be estimated through the integration method but based on the ignore RT distribution, which is the method we used in this case. Still, it is worth noting that this solution is only valid as long as the assumption of equal slowing holds, and therefore, the SSRTs computed using the ignore RT distribution should be interpreted with caution.

Post-stop error and post-ignore slowing. We compared the RT of go trials following either a failed stop trial or a correct ignore trial, with the RT of go trials that happened after a correct go trial (in the task sequence, this “go after go” trial is the one that precedes the error with greatest proximity). This method prevents confounds stemming from participants’ changes in ability, motivation or response caution throughout the task [51]. Additionally, post-stop success slowing was also calculated following the same procedure in order to have a control condition with minimal conflict and without errors.

Premature responses. These were computed as those emitted before the presentation of the go stimulus on a go trial. Importantly, we discounted responses considered as atypically slow from this measure. If a response was emitted before the go stimulus but, at the same time, no response was emitted in the previous trial, we considered this sequence as a slow response to the previous trial instead of a premature response to the current trial.

#### 2.3.2. Attention-Related Measures

Intra-individual variability. This refers to a participant’s variation in performance of reaction times across trials within the task, in part due to attentional lapses [38,39,40]. The response variability of each participant was estimated by fitting their RT to an ex-Gaussian distribution. This distribution is formed by the convolution of a normal and an exponential distribution, and it is characterized by three parameters: the mean and the standard deviation of the normal component (mu and sigma, respectively), and the mean and the standard deviation of the exponential component (both described by tau). Importantly, the ex-Gaussian distribution was not fitted to the complete go RT distribution, given that stop–go and ignore–go trial sequences would introduce proactive or post-response adjustments that probably bias response variability. Thus, we selected only those go trials that followed another go trial for this analysis. This fitting process was analysed in the DISTRIB toolbox for MATLAB [40].

Go and ignore omissions. A go omission is defined as a go trial without a response, whereas an ignore omission is an ignore trial without a response.

### 2.4. Data Analyses

Data distributions were examined for outliers and normality within each group. Outliers were identified as those data points further than 1.5 times from the upper/lower limit of the interquartile range for each quantitative variable, and then, were replaced by the mean ± 2 standard deviations of the corresponding group. The number of outliers only represented 0.01% of the dataset (10/1200; 6 in the older group and 4 in the younger group). Normality was assessed by using the Kolmogorov–Smirnov test and by examining normal QQ plots of the data. Where the distributions were normal, parametric tests were used; otherwise, non-parametric tests were performed. Given that the obtained results were equivalent using parametric and non-parametric tests, we report the former for all measures in-text to simplify presentation of the results. In any case, the results of the non-parametric tests can be found in the Supplementary Materials (Supplementary Table S1).

Contingency tables were used to examine whether the ratio of strategy choice varied between age groups. Since we expected that certain strategies would be chosen by few participants, the Freeman–Halton extension of Fisher’s exact test was used instead of a chi-squared test [52]. Cramer’s V was used as a measure of effect size. Follow-up Z-tests for independent proportions were performed to determine significant differences between groups (*p* < 0.05, 2-tailed, Bonferroni corrected).

Group differences in quantitative measures of the stimulus-selective stopping task were assessed using a multivariate analysis of covariance (MANCOVA) with group as the between-subjects factor and the strategy adopted to complete the task as the covariate. Follow-up one-way ANCOVAs were conducted. To avoid type I error by multiple comparisons (*n* = 10), we used a more conservative threshold of significance (*p* < 0.01). Effect sizes were calculated using partial eta-squared (ηp^2^). As a rough guideline, an ηp^2^ of 0.01 constitutes a small effect, 0.06 a medium effect and 0.14 a large effect [53].

Lastly, we performed a logistic regression model using the backward stepwise method (with AIC selection for removal of predictors) to assess which task-related measures could be considered significant predictors of age group status. The strategy and all quantitative measures showing significant differences between groups in previous analyses (ANCOVAs) were included in the starting model. We report the results of the final step of the regression. The performance of the model was assessed using the Area Under the ROC Curve (AUC). Analyses were performed using SPSS 26 (Contingency tables and MANCOVA-ANCOVAs) (SPSS Inc., Chicago, IL, US) and JASP 0.11.1 (logistic regression; JASP, 2019) unless mentioned otherwise. Data from this study are available in the figshare repository (https://doi.org/10.6084/m9.figshare.14510490.v1).

## 3. Results

The strategy followed by each participant was estimated by comparing their mean no-signal (go) RT, stop-respond RT and ignore RT following the procedure used by Bissett and Logan [16], as explained before. Twenty participants used the non-selective StD strategy, whereas ninety-two adopted selective strategies (80 iDtS and 12 dDtS). The strategy used to solve the stimulus-selective stop-signal task could not be estimated in 21 participants (14.7% of the total sample: 10 younger children and 11 older children). Means and standard deviations of no-signal (go), stop-respond (failed inhibition) and ignore RTs, as well as of SSRT and the SSD, as a function of group and strategy are shown in Table 1.

The probability of responding on the stop trials (respond|signal) did not differ between groups (0.49 ± 0.06 and 0.51 ± 0.07 for younger and older children, respectively; t (118) = −1.81, *p* = 0.07). By contrast, as expected, mean SSD was longer for younger than for older children (298.83 ± 103.93 and 250.44 ± 121.4, respectively; t (118) = 2.35, *p* = 0.02, Cohen’s d = 0.43).

**Figure 2:**
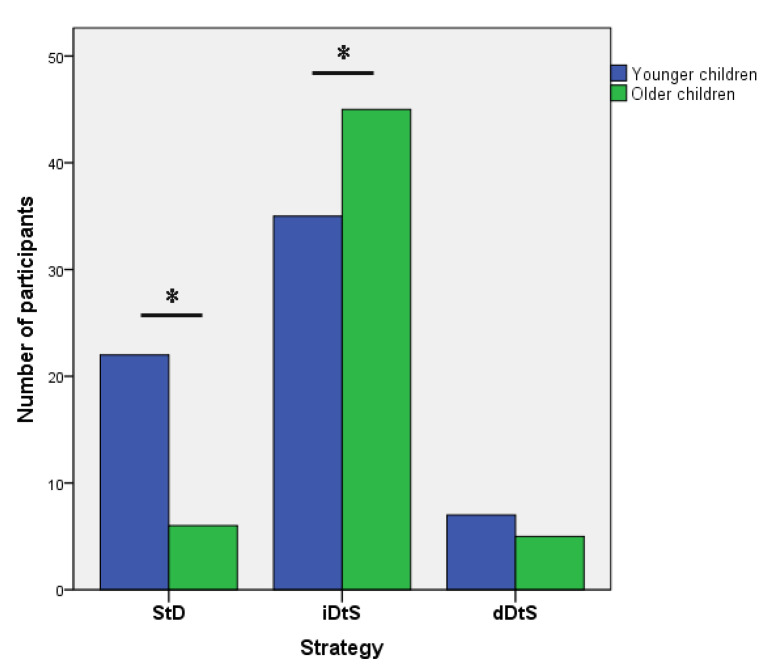
Number of participants adopting each strategy in each age group. StD = Stop then Discriminate strategy; iDtS = Independent Discriminate then Stop strategy; dDtS = Dependent Discriminate then Stop strategy. Asterisk denotes significant differences between younger and older children.

Strategy and age group were associated (Fisher’s exact test = 10.42, *p* = 0.004, Cramer’s V = 0.29; Figure 2). Z-tests for independent proportions showed that the proportion of children using the StD strategy was significantly higher in the younger (34.4%, *n* = 22) than in the older group (10.7%, *n* = 6). By contrast, the proportion of children using the iDtS strategy was significantly lower in the younger (54.7%, *n* = 35) than in the older group (80.4%, *n* = 45). We observed no significant differences in the adoption of dDtS strategy between younger and older children (10.9%, *n* = 7 vs. 8.9%, *n* = 5, respectively).

**Figure 3:**
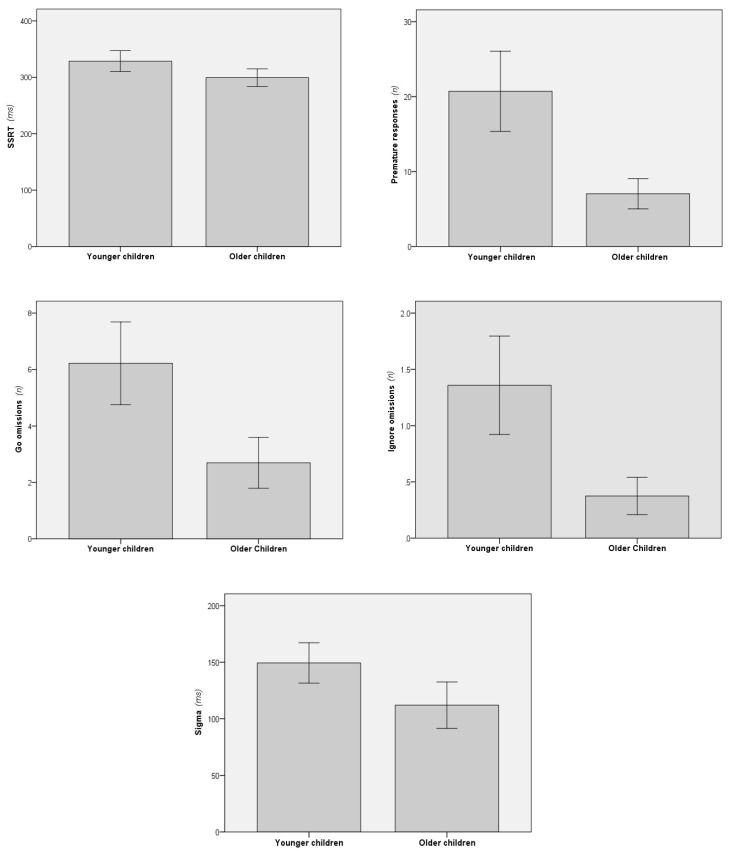
Quantitative measures of the stimulus-selective stopping task showing significant differences between groups. Error bars denote 95% confidence interval. SSRT = stop-signal reaction time.

Group differences in quantitative measures of the stimulus-selective stopping task were firstly assessed using a MANCOVA with group as the between-subjects factor and the strategy as the covariate. It revealed a significant main effect of group (F (10, 108) = 5.56, *p* < 0.001, ηp^2^ = 0.34). The effect of strategy was also significant in the model (F (10,108)) = 3.29, *p* < 0.001, ηp^2^ = 0.23), but follow-up ANCOVAs showed that it was restricted to SSRT (F (1,117)) = 23.24, *p* < 0.001, ηp^2^ = 0.17). Thus, the follow-up one-way ANCOVA revealed that SSRTs were longer in younger than in older children, even after controlling for the confounding effects of strategy adoption (F (1,117) = 9.78, *p* = 0.002, ηp^2^ = 0.08; Figure 3). Differences between age groups were also found in the number of premature responses, go and ignore omissions and sigma (Figure 3). Means and standard deviations of all quantitative variables for both age groups are also shown in Table 2, along with the F and *p* values from the ANCOVAs.

Finally, logistic regression analyses using the backward stepwise method showed that out of all the variables entered in the model, only ignore omissions, premature responses, and the strategy predicted age group status. The results for the last step of the regression can be seen in Table 3. The AUC value of the model was 0.837, indicating good performance.

## 4. Discussion

This study provided some novel insights about the development of selective inhibitory control in middle childhood using a stimulus-selective stop-signal task. We hypothesized that selective strategies (iDtS and dDtS) would be more likely to be used by older children. In this sense, we found the iDtS strategy to be the most used both in younger and older children. Even so, there was a significant increase in its use in older children compared to younger children, so that very few children used the StD strategy by the end of middle childhood. Selective inhibition is more complex than global inhibition due to the participation of additional inhibitory processes (both reactive and proactive) and also due to the greater involvement of other cognitive processes such as working memory and perceptual and attentional discrimination [16,17,19,41]. This greater complexity is also evidenced by the recruitment of additional brain areas, such as the dorsolateral prefrontal cortex, the insula or the striatum [17,18,19,54], which suggests that the level of brain maturation required for proper selective inhibitory control is presumably achieved slightly later than for global inhibition. Although all strategies can be used to achieve task demands, it could be argued that the use of the StD strategy may be less adaptive in some real-world situations, since it involves stopping indiscriminately to all stimuli and then restarting the response to those stimuli that required it. This remains an open question that future studies will need to examine.

It should be noted that we did not observe any significant differences between younger and older children in the use of the dDtS strategy. As seen in a previous study with adults, this selective strategy seems to be used only by a small number of participants when discrimination between stimuli is relatively easy [41], and is characterized by the fact that the need to discriminate between signals produces an interaction between the stopping and the responding processes that violates the assumptions of the horse race model [16,25]. In addition, our study suggests that the adoption of this strategy is infrequent across all ages, at least when signal discrimination difficulty is low.

Notably, in the current study, strategy is a predictor of age group status in our logistic regression analyses. In this sense, the fact that the strategy adopted by the participants is one of the predictor variables in the regression model and the SSRT is not suggests that the way in which participants achieve task goals in the stimulus-selective stop-signal task (selectively or non-selectively) seems to be one of the most notable changes in inhibitory control at this developmental stage. Nonetheless, our findings point to the question of whether the decrease in adoption of the non-selective strategy extends beyond middle childhood, or if middle childhood represents the main critical stage for the maturation of selective inhibitory control.

Results regarding the SSRT are in accordance with our hypotheses and showed that, once the effect of strategy was controlled for, the SSRT was shorter for older children compared to younger children. This finding suggests a greater inhibitory efficiency in older children independent of the type of inhibition used (selective or global), which is in line with previous studies using selective stopping paradigms that did not control for strategy adoption [12,36,37] and also in line with studies of global stopping [12,55]. Further research with larger sample sizes is needed to investigate whether developmental differences in the SSRT are similar or different in selective and non-selective (global) strategies.

With respect to post-signal response adjustments, both younger and older children slowed down after committing an error in a stop trial and after a correct response in an ignore trial (the slowing was similar between stop–error and correct–ignore conditions across participants, results not presented above). By contrast, after successfully stopping, participants did not need to slow down. Thus, cognitive control (including some degree of proactive inhibitory control) is involved in both post-stop error and post-ignore slowing. However, it should be noted that we did not find a reduction in the degree of post-stop error and post-ignore slowing between age groups. Therefore, younger and older children were presumably applying equivalent degrees of proactive control to prevent further errors and/or resolve conflict. This finding is in agreement with previous literature [44,45], albeit some studies have found changes in post-error slowing during this developmental period (see [44] for a review). This discrepancy might be due in part to the way we calculated post-error (and post-ignore) slowing, using a method that prevents confounds [51] and that has usually not been applied in previous studies. Furthermore, factors intrinsically related to task design and that vary from task to task might explain the mixed findings and interpretations [44,45]. Further studies are needed to examine whether the maturation of the proactive control reflected in these post-signal adjustments happens before or after middle childhood.

Our analyses regarding premature responses also offer some insights. Premature responding is another motoric form of impulsivity [32,33,34], where a tendency to respond before stimulus presentation denotes a greater “waiting” impulsivity. Whereas stopping impulsivity (SSRT) is thought to be mainly mediated by the dorsal frontostriatal circuit, waiting impulsivity (premature or anticipatory responding) seems to be primarily supported by the ventromedial frontostriatal network [34]. The fact that younger children made more premature responses than older children suggests that middle childhood is a critical period not only for “stopping impulsivity”, but also for “waiting impulsivity”. Remarkably, premature responding was also one of the predictors of group membership in our logistic regression analyses.

Regarding attention, we observed changes in intra-individual variability that are partially in line with previous studies. Using other inhibitory tasks such as the go/no-go or the flanker task, intra-individual variability has been shown to decrease as children grow older, accompanied by maturation of both the frontoparietal-thalamic network and of white matter integrity [56,57]. When intra-individual variability is measured through sigma and tau parameters of the ex-Gaussian distribution of reaction times, the former is thought to capture the variability in fast responses, while the latter would reflect the variability in slow responses, which is thought to be related to greater lapses in attention [58]. In partial agreement with our hypotheses, we observed a reduction in the sigma parameter throughout middle childhood, which replicates previous findings from other tasks [57,58]. However, we did not find any differences in the tau parameter, which was unexpected since a reduction in tau with age has been found in typically developing children at this stage [58,59,60]. This might be due to the fact that we used short ISIs in our task. In this sense, a recent study showed that the tau effect (intermittent lapses in attention) might be more noticeable when longer ISIs are used, probably because a higher attentional demand is placed on the participants [61].

As for go and ignore response accuracy, both omissions to the go stimulus and omissions to the ignore stimulus showed differences in younger and older children, with younger children making more errors. This is in accordance with our hypotheses and with previous studies [60,62], and suggests that the ability to focus and sustain attention on the ongoing task develops with age. Notably, only ignore omissions were a predictor of age group in our logistic regression analyses. In contrast to a go omission, which implies ignoring only the go stimulus, an ignore omission involves disregarding both the ignore signal and the go stimulus in the same trial. Thus, it could be argued that ignore omissions are a sign of greater distractibility than go omissions. Of note, this attentional measure derived specifically from ignore trials cannot be calculated using other tasks, and seems to be an interesting marker of attentional maturation at this developmental stage.

There are some limitations in our study that should be taken into account. Firstly, this is a cross-sectional study, and thus, our conclusions should be further confirmed by a longitudinal approach that follows each child along their development to examine changes in their strategy choice and in quantitative task-related measures. Secondly, given the rather low percentage of use of StD and, particularly, dDtS strategies, it would be interesting to explore whether it is possible that SSRT differences between younger and older children might be greater in non-selective or in selective strategies. This would require larger sample sizes with a representative number of participants per strategy. Thirdly, our study focuses on middle childhood years, but it would be of interest to explore any possible further changes in selective inhibitory control, both in earlier stages of maturation (early childhood) and during adolescence. In adulthood, a recent study did not find differences in the adoption of strategies between 24 young adults (20–30 years) and 24 older adults (61–76 years) [63]. Finally, our study focuses on the strategies as a behavioural measure, but future research would benefit from exploring in depth the neural correlates of each strategy and its relation to the maturation of several brain networks in middle childhood.

## 5. Conclusions

The results of our study show that most children were able to interrupt their ongoing responses selectively at the end of middle childhood. We also observed a strategy-independent improvement in efficiency to cancel motor responses (as indexed by faster SSRTs) during this developmental stage. Additionally, by using the stimulus-selective stopping task, we were able to examine other forms of inhibitory control/impulsivity such as premature or anticipatory responding and post-stop error slowing, task-specific factors (ignore omissions and post-ignore slowing) and well-established attentional measures (go omissions and intra-individual variability). Overall, our data indicate that middle childhood is a milestone in the development of crucial aspects of motor inhibition, which possibly reflects the progressive maturation of an extensive network of brain areas underlying selective and proactive stopping that goes beyond those involved in global and reactive stopping.

## Figures and Tables

**Table 1:** Means and standard deviations of reaction times by group and strategy.

	Younger Children						Older Children					
	StD		iDtS		dDtS		StD		iDtS		dDtS	
	Mean	SD	Mean	SD	Mean	SD	Mean	SD	Mean	SD	Mean	SD
Go RT	671.32	110.70	685.89	137.80	611.06	73.56	561.02	131.18	587.11	147.38	565.00	116.35
Ignore RT	779.45	128.25	721.02	148.57	690.11	107.49	648.75	140.72	600.75	149.42	676.31	158.97
Failed stop RT	516.65	104.89	521.24	103.39	571.34	85.39	433.47	96.31	442.52	102.84	522.48	97.87
SSRT *	314.14	68.70	334.85	76.09	439.41	134.01	281.20	64.84	297.40	49.04	449.72	71.84
SSD	302.27	105.20	309.29	107.80	235.71	59.26	245.83	108.88	252.78	127.28	235.00	96.18

* Note: the SSRT was calculated using the go RT distribution in the StD and iDtS strategies, and using the ignore RT distribution in the dDtS strategy. Abbreviations: StD = Stop then Discriminate strategy; iDtS = Independent Discriminate then Stop strategy; dDtS = Dependent Discriminate then Stop strategy; SD = Standard Deviation; RT = Reaction Time; SSRT = Stop-Signal Reaction Time; SSD = Stop-Signal Delay.

**Table 2:** Means, standard deviations and ANCOVA results for all quantitative variables.

	Younger Children		Older Children		ANCOVAs		
	Mean	SD	Mean	SD	F (1117)	Uncorrected *p* Value	*η*_p_^2^
SSRT	339.17	88.06	309.26	68.38	9.78	0.002 *	0.08
Premature responses	20.70	21.41	7.04	7.52	23.10	<0.001 *	0.17
Mu ^1^	471.72	143.26	410.68	140.18	5.37	0.022	0.04
Sigma ^2^	149.34	71.58	112.02	76.46	7.26	0.008 *	0.06
Tau ^3^	158.57	87.80	142.77	79.45	0.66	0.417	0.01
Go omissions	6.22	5.86	2.70	3.38	16.32	<0.001 *	0.12
Ignore omissions	1.36	1.75	0.38	0.62	17.55	<0.001 *	0.13
Post-stop success	4.61	145.11	1.02	125.17	0.09	0.76	0.01
Post-stop error	130.53	146.21	100.73	115.31	1.57	0.21	0.01
Post-correct ignore	167.45	207.40	109.88	79.81	3.78	0.05	0.03

Note: error data are given as a total number, not as a rate.* Significant with a more conservative threshold *p* value of 0.01.1; ^1^ Mu is the mean of the normal component of the ex-Gaussian distribution, and it is thought to reflect processing speed; ^2^ Sigma is the standard deviation of the normal component of the ex-Gaussian distribution, and it is thought to be a measure of variability; ^3^ Tau is the mean and standard deviation of the exponentially distributed tail of the distribution, and it is often associated with lapses of attention. Abbreviations: SD = Standard Deviation, SSRT = Stop-Signal Reaction Time.

**Table 3:** Logistic regression results.

	Heading			Wald Test		95% Confidence Interval	
	Beta	Standardized Beta	Odds Ratio	Wald Statistic	*p*	Lower Bound	Upper Bound
Ignore omissions	−0.63	−0.91	0.53	6.24	0.01	−1.13	−0.14
Premature responses	−0.07	−1.23	0.93	8.68	0.003	−0.12	−0.02
Strategy (iDtS)	2.17	2.17	8.72	14.28	<0.001	1.04	3.29
Strategy (dDtS)	1.56	1.56	4.74	3.45	0.06	−0.09	3.20

Note: older children coded as class 1. StD strategy used as reference. Sensitivity of the model = 0.79. Specificity of the model = 0.78.

## Data Availability

The data presented in this study are available in the present paper, in its Supplementary Materials, and openly available in https://doi.org/10.6084/m9.figshare.14510490.v1.

## Supplementary Materials

The following are available online at https://www.mdpi.com/article/10.3390/ijerph18126300/s1, Table S1.
