# Supplementary material for: Selective Inhibitory Control in Middle Childhood
Source: Int J Environ Res Public Health. 2021 Jun 10;18(12):6300. doi: 10.3390/ijerph18126300 (PMC8296104; doi:10.3390/ijerph18126300)
Supplement: Supplementary file 1 [file ijerph-18-06300-s001.zip › ijerph-1223016-supplementary.pdf]

Table S1. Non-parametric (Mann-Whitney) results for quantitative variables not normally distributed[1].

	Mann Whitney	
	Z	Uncorrected *p* value
**Premature responses**	-4.81	< 0.001*
**Mu[2]**	-2.88	0.004*
**Sigma**	-3.07	0.002*
**Go omissions**	-3.96	< 0.001*
**Ignore omissions**	-3.31	< 0.001*
**Post-correct ignore**	-2.37	0.02

*[1]SSRT, post-stop success, post-stop error and tau were normally distributed according to Kolmogorov-Smirnov tests. [2]Mu was the only variable that showed significant differences between groups in the nonparametric tests but not in the parametric tests. Mu is not related to inhibitory control. It is the mean of the normal component of the ex-Gaussian distribution, and it is thought to reflect processing speed. *Significant with a more conservative threshold p value of 0.01*
